# Systematic Aetiological Assessment of Myocarditis: A Prospective Cohort Study

**DOI:** 10.3390/jcm13041025

**Published:** 2024-02-10

**Authors:** Vincent Michel, Estibaliz Lazaro, Thomas Fauthoux, Laura Cetran, Cécile Contin-Bordes, Patrick Blanco, Benjamin Seguy, Thomas Baudinet, Pierre Coste, Edouard Gerbaud

**Affiliations:** 1 Cardiology Intensive Care Unit and Interventional Cardiology, Hôpital Cardiologique du Haut-Lévêque, 33604 Pessac, France; vincent.michel@chu-bordeaux.fr (V.M.); laura.cetran@chu-bordeaux.fr (L.C.); benjamin.seguy@chu-bordeaux.fr (B.S.); thomas.baudinet@chu-bordeaux.fr (T.B.); pierre.coste@u-bordeaux.fr (P.C.); 2 Service de Médecine Interne, Centre de Référence des Maladies Auto-Immunes Systémiques Rares de l’Est et du Sud-Ouest, Bâtiment des USN, Hôpital du Haut-Lévêque, 33604 Pessac, France; estibaliz.lazaro@chu-bordeaux.fr (E.L.); thomas.fauthoux@hotmail.fr (T.F.); 3 CNRS-UMR 5164, ImmunoConcept, Université de Bordeaux, 33076 Bordeaux, Cedex, France; cecile.bordes@chu-bordeaux.fr (C.C.-B.); patrick.blanco@chu-bordeaux.fr (P.B.); 4 Laboratoire d’Immunologie et Immunogénétique, FHU ACRONIM, Hôpital Pellegrin, Centre Hospitalier Universitaire, 33076 Bordeaux, France; 5 Bordeaux Cardio-Thoracic Research Centre, U1045, Bordeaux University, 33076 Bordeaux, France

**Keywords:** myocarditis, autoimmune and inflammatory disorders, aetiological assessment

## Abstract

Background: Myocarditis is commonly diagnosed in the intensive care cardiology unit (ICCU). No current recommendation nor guideline aids exist for aetiological assessments. Methods: From September 2021 to October 2023, 84 patients with acute myocarditis underwent thorough and systematic serum and blood cell panel evaluations to determine the most common causes of myocarditis. Results: Of the 84 patients (median age 34 years, range 22–41 years, 79% male), 16 presented with complicated myocarditis. The systematic aetiological assessment revealed that 36% of patients were positive for lupus anticoagulant, 12% for antinuclear antibodies, 8% for anti-heart antibodies, and 12% for anti-striated muscle antibodies. Viral serology did not yield any significant results. After the aetiological assessment, one patient was diagnosed with an autoimmune inflammatory disorder (Still’s disease). T-cell subset analyses indicated that myocarditis severity tended to increase with the T-cell lymphopenia status. Conclusions: A comprehensive, systematic aetiological assessment was of limited value in terms of predicting the clinical or therapeutic outcomes in myocarditis patients presenting to the ICCU.

## 1. Introduction

Acute myocarditis has become well-recognised by clinicians; the incidence is steadily approaching 10 per 100,000 subjects [1,2]. Acute myocarditis is a clinical diagnosis. The clinical presentation of acute myocarditis is highly variable, ranging from asymptomatic or mild febrile illness to cardiogenic shock and sudden cardiac death. Acute myocarditis is generally identified by the sudden onset of chest pain and dyspnea. The initial presentation may be acute or insidious in onset and progression. There are no pathognomonic clinical features. Patients may also present with fever, malaise, fatigue, presyncope, or syncope. The chest pain of acute myocarditis can mimic the pain of pericarditis or can occasionally be severe central pain mimicking the pain of acute coronary syndrome. Diagnosis has been significantly improved by advances in cardiac magnetic resonance imaging (CMRI), which affords non-invasive confirmation of the condition and enables assessment of the disease burden by quantifying cardiac damage [3]. Despite this, certain specific indications for endomyocardial biopsies (EMBs) remain for patients with complicated myocarditis. EMBs play crucial roles in defining targeted treatment strategies based on the histopathological profiles [4,5]. Many factors trigger myocarditis, including viral and bacterial infections, autoimmune and inflammatory disorders, and adverse reactions to drugs and vaccines [6,7,8,9]. Recent investigations have explored the intricate interplay among genetic factors in terms of myocarditis development. Notably, 5–8% of patients with acute myocarditis exhibit truncated genetic variants associated with dilated or arrhythmogenic cardiomyopathy [10]. Analysis of the Lombardy registry cohort of patients with autoimmune and inflammatory diseases (AIIDs) revealed that 7.2% of such patients are affected; this figure rises to 15.2% in those with complicated myocarditis [11]. 

However, these advances have not found applications in clinical practice. Presently, no consensus or recommendation that guides the systematic exploration of aetiological factors is available. Physicians engage in medical history taking and review the clinical presentation. To the best of our knowledge, no study has specifically explored the utility of a systematic, non-invasive aetiological evaluation of myocarditis that includes potential viral and immunological causes. Thus, we used a standardised assessment protocol for the systematic exploration of the aetiological profile of acute myocarditis. The various known causes of the condition were included in the evaluation. The immunological profiles of patients with acute myocarditis were examined, and it was hypothesised that myocarditis sometimes indicates the commencement of a more systemic disease. 

## 2. Materials and Methods

### 2.1. Study Population

We performed a prospective, single-centre study of all consecutive patients admitted to our intensive care cardiology unit (ICCU) from September 2021 to October 2023, with a confirmed diagnosis (by CMRI and/or EMB) of acute myocarditis. The admission criteria to the ICCU included all patients presenting with myocarditis, provided that cardiac symptoms were predominant or required monitoring in a specialized environment. CMRI was performed during hospitalisation using a 1.5 Tesla system (Magnetom Avanto, Siemens Medical Systems, Erlangen, Germany). The CMRI protocol included cine-imaging, T2-weighted first-pass perfusion, conventional breath-hold late gadolinium enhancement (LGE), and high-resolution LGE (HR-LGE) under the free-breathing condition. The left ventricular ejection fraction was calculated using Argus software (Siemens Medical Systems). CMRI-based diagnosis was based on the updated 2018 Lake Louise criteria for acute myocarditis [3]; the European Society of Cardiology criteria were used when evaluating EMB data [8]. Complicated myocarditis was defined as myocarditis with a left ventricular ejection fraction <50% on the first echocardiogram, sustained ventricular arrhythmias, and/or low cardiac output syndrome [5,11]. 

### 2.2. Systematic Aetiological Evaluation

All blood samples collected during the ICCU stay were subjected to comprehensive analyses. We assayed the levels of antinuclear antibodies (ANAs) and anti-extractable nuclear antigen (ENA) in ANA-positive patients (ANA was defined as positive in titre ≥1:160); anti-RNA-pol III, anti-dsDNA, and antiphospholipid antibodies; anti-neutrophil cytoplasmic antibodies (ANCAs); anti-myocardium antibodies; and anti-striated muscle antibodies. We performed T-lymphocyte phenotyping. ANAs were detected using an indirect immunofluorescence (IFI) assay that employed HEp2 cells (Nova Lite; Werfen; Barcelona; Spain). Anti-ENA (including anti-Sm, anti-ribonucleoprotein, anti-SSA, anti-SSB, anti-centromere B, anti-Scl-70, anti-Jo-1, and anti-dsDNA) antibodies were quantitated using chemiluminescence assays on the Bioflash system (Werfen). The cutoff levels were 20 CU/mL for anti-ENA and 35 IU/mL for anti-dsDNA. Anti-RNA pol III antibodies were detected using the EliA Immunocap system (Thermo Fisher, Waltham, MA, USA) at a positive cutoff level of 5 AU/mL. ANCAs were measured using the standard IFI assay that employs ethanol-fixed group O human neutrophils (51/40 screen dilution). Anti-proteinase 3 antibody and myeloperoxidase levels were measured using chemiluminescence assays on the Bioflash apparatus (Werfen); the cutoff level was 20 CU/mL. The antiphospholipid antibody assays measured the levels of lupus anticoagulant, anticardiolipin IgG/IgM antibodies, and anti-β2 glycoprotein I IgG/IgM antibodies. Lupus anticoagulant was detected by a functional coagulation assay; anti-β2 glycoprotein I antibodies were quantified using an enzyme-linked immunosorbent assay (ELISA) and anticardiolipin antibodies were found using a chemiluminescence assay. Anti-myocardium antibodies and anti-striated muscle antibodies were determined using IFI on a monkey heart and skeletal muscle, respectively, using a commercial kit (EuroImmun) at 1/100 serum dilution, according to the manufacturer’s instructions. T-lymphocyte phenotyping was performed by flow cytometry using the Becton Dickinson FACsCanto platform. All antibodies (anti-CD3, CD4, CD8, and HLA-DR) were from Becton Dickinson. Various infectious agents including HIV, HBV, HCV, Epstein–Barr virus (EBV), CMV, and the Lyme disease pathogen were screened using an ELISA blood test. The minimum standard aetiological blood panel was used to investigate the acute myocarditis status established by the internal medicine team of our hospital.

All patients were systematically encouraged to undergo thoracic imaging, and we evaluated potential indicators of systemic disease. All patients underwent follow up by an internist within 3 months thereafter. Complete hospital data were collected using DxCare software (Medasys). The systematic panel was complemented with additional tests indicated by the clinical context. These were at the discretion of the cardiologist and internist who reviewed all of the patients during the consultations.

### 2.3. Outcomes

The Six-month follow-up outcome data were gathered by referencing the medical records or by directly contacting the patients.

### 2.4. Statistical Analysis

All data were anonymised prior to analysis. Categorical variables are presented as frequencies or percentages and continuous variables as medians with interquartile ranges unless otherwise stated. All data distributions were tested in terms of normality, and appropriate (parametric or non-parametric) tests were then chosen. Continuous variables were compared using Student’s *t*-test or the non-parametric Mann–Whitney U test. Categorical variables were compared using the χ^2^ test with the Yates correction. *p*-values < 0.05 were considered significant. All analyses were conducted using NCSS 2001 Statistical Software (NCSS, Kaysville, UT, USA). 

### 2.5. Ethics

Data were prospectively collected without implementation of any supplementary therapeutic interventions or extra monitoring procedures. In accordance with French legislation, the absence of any such interventions rendered approval by an independent ethics committee unnecessary. Informed consent was carefully obtained from all participants. 

## 3. Results

### 3.1. Population

From September 2021 to October 2023, 84 consecutive patients diagnosed with acute myocarditis were admitted to the ICCU of Bordeaux University Hospital, of whom 16 (19%) exhibited complicated myocarditis. Of these sixteen patients, five (31%) required vasopressors and/or inotropic agents. No patients were presented with sustained ventricular arrhythmias.

As indicated in Table 1, most of the patients were young males who presented with the common clinical symptoms of chest pain and a recent history of flu-like syndrome. In terms of medical history, only four patients had a prior diagnosis of myocarditis, and only a few patients had a history of chronic infectious or inflammatory disease. Additionally, one patient was undergoing treatment for an HIV infection; another was receiving therapy for rheumatoid arthritis; and a third was undergoing diagnostic workup for a prolonged febrile illness.

Overall, four patients were on current immune checkpoint inhibitor treatments with nivolumab, adalimumab, tafinlar/mekinist, or dostarlimab/anti-TIGIT. Of these, three (75%) patients were presented with complicated myocarditis. In terms of the genetic background, one patient had a history of von Hippel–Lindau disease and another patient had a history of inherited cardiomyopathy associated with a mutation in the phospholamban gene.

### 3.2. Imaging and Diagnostic Modalities

CMRI was performed within the initial 7 days of hospitalisation for 83 cases; their primary characteristics are detailed in Table 2. EMB was conducted in only five patients, all of whom had complicated myocarditis. Of these, only one had an EMB profile (a giant cell pattern) consistent with a diagnosis of myocarditis. The remaining four EMBs exhibited non-specific findings, including discrete lymphocytic infiltrates that did not meet the diagnostic myocarditis criteria. Overall, three of the five biopsies were positive by PCR for parvovirus B19.

Pericardial effusion was infrequently observed in the initial transthoracic echocardiography of the patients, with only two cases in the complicated group and one case in those with uncomplicated myocarditis. A coronary assessment was performed in 34 patients (40%) using either computed tomography (CT) or, more commonly, coronary angiography (CA). These did not reveal any significant coronary artery disease, except in one patient with a tight lesion at the ostium of the posterior retroventricular branch. 

### 3.3. Outcomes and Follow Up

Table 3 summarises the outcomes during the hospital stay and the discharge medications prescribed by the attending cardiologist. It is worth noting that the patient requiring extracorporeal life support achieved a full recovery and did not require a heart transplantation. The two patients who died were not eligible for heart transplantation.

Of the 82 patients who survived after discharge, the follow-up data at 6 months were successfully gathered for 58 patients. Of the other 24 patients, 11 had not yet reached the 6-month milestone, and 13 were lost to follow up. Of the 58 patients with available follow-up data, four experienced recurrent chest pain by 6 months. Notably, all patients exhibited a preserved ejection fraction on transthoracic echocardiography or CMRI. Of the 49 patients who underwent exercise testing, two displayed pathological ventricular arrhythmias. Of the 22 patients who underwent CMRI, 18 (82%) evidenced persistent delayed enhancement, i.e., fibrotic scars.

### 3.4. Systematic Aetiological Assessment

Standard blood panel data were collected at ICCU admission to treat chest pain or dyspnoea. Table 4 lists the baseline biological characteristics of the 84 patients. 

Compared with patients with uncomplicated myocarditis, patients with complicated myocarditis exhibited elevated troponin and creatinine kinase levels and greater inflammation, as indicated by higher WBC counts and CRP levels. The infectious panel tests did not yield any significant findings. While one patient was positive for HIV, such seropositivity was already known, and the viral load was undetectable. Additionally, two patients were PCR-positive for COVID-19. Another patient had recently suffered a primary EBV infection. Also, two patients with respiratory symptoms exhibited an elevated blood level of anti-*Mycoplasma pneumoniae* IgM. Lyme disease serological tests were positive in two patients, but the results lacked clinical significance given the absence of any compatible medical history. Overall, three of five viral PCR tests conducted in EMB samples were positive for parvovirus B19.

Patients with myocarditis exhibited distinctive immunological profiles characterized by frequent positive test results for non-specific ANAs and a distinct antiphospholipid antibody signature, including lupus anticoagulant positivity (Table 5). Despite the presence of RNA pol III antibodies in one patient, a thorough evaluation by the internal medicine team did not yield a diagnosis of scleroderma. Of the patients with anti-myocardium antibodies, only one exhibited a discernible finding, specifically an intercalated disc. 

As part of the systematic assessment, we examined the T-lymphocyte subset status; the findings are presented in Table 6.

Patients with complicated myocarditis exhibited significantly more global T-cell lymphopenia than did those with uncomplicated myocarditis (*p* = 0.001). Such lymphopenia included both CD4+ and CD8+ T lymphocytes. Patients with complicated myocarditis requiring vasopressors tended to exhibit more CD8+ T-cell lymphopenia compared with those with uncomplicated myocarditis who did not require vasopressors (CD4+/CD8+ T-cell ratio: 2.9 in vs. 2.0; *p* = 0.29). One patient with complicated myocarditis exhibited lymphocytosis that involved Vδ2 T cells, despite the absence of any specific infection typically associated with such cells.

Thoracic imaging using either CT or positron emission tomography–CT was performed in 64 patients (76%). Abnormalities warranting further investigation were found in 17 (27%), of whom five underwent lymph node biopsies. However, these complementary investigations did not yield any insight into the aetiology of myocarditis. 

A total ofsixty-nine patients (82% of the total) attended consultations with the internal medicine team. Eventually, aetiological diagnoses were established for six of the sixteen patients with complicated myocarditis, including one case of giant-cell myocarditis, two cases of immune checkpoint inhibitor myocarditis, one case associated with cocaine use, one case linked to a phospholamban mutation, and one case linked to COVID-19 infection. Of the non-complicated myocarditis cases, three were attributable to post-COVID-vaccine myocarditis, one to COVID-19 infection, one to primary EBV infection, and one to recently discovered Still’s disease.

## 4. Discussion

In this comprehensive study, we meticulously characterised consecutive cases of myocarditis encountered by cardiologists working in an ICCU, with a specific focus on aetiological aspects that might be revealed using a systematic approach. The demographics and clinical presentations of our patients resembled those of larger contemporary myocarditis cohorts. A primary finding was that the systematic aetiological assessment did not reveal any specific infectious, autoimmune, or inflammatory disease that caused myocarditis. However, the level of anti-phospholipid antibodies was elevated during the acute phase, and this elevation persisted for some time in certain patients. Also, notable trends in the T-lymphocyte subset profiles were apparent and were correlated with disease severity.

The demographic characteristics of our study population were very similar to those of previously reported larger myocarditis cohorts in terms of age, sex, and clinical presentation. This not only validates the representative nature of our study but also facilitates a nuanced analysis of myocarditis within the specific context of cardiac intensive care. Notably, the incidence of complicated myocarditis in our study (19%) is very close to those of recent studies, reinforcing the reliability of our observations [11]. A distinctive feature of our study population was the rarity of any AIID history. This differs from the situation in other myocarditis cohorts; for example, the Lombardy cohort exhibited a 7.2% prevalence of associated autoimmune disorders [11]. The difference is likely attributable to our recruitment strategy. We included only ICCU patients; thus, no patients from internal medicine or rheumatology wards that often treat patients with AIIDs were included. As previously reported [12], the diagnostic utility of EMB of the right ventricle alone was suboptimal in terms of sensitivity; only one biopsy met the recognised diagnostic criteria. The remaining four biopsy specimens exhibited inflammatory patterns below the currently accepted threshold, thereby exacerbating sampling accuracy concerns. Parvovirus B19 PCR tests were positive in three of the EMB cases, adding to the ongoing controversy of the role played by parvovirus B19 in acute myocarditis [13]. 

Regarding the systematic aetiological assessment, as reported previously [14], serological analysis of viral infection did not aid in the diagnosis of myocardial infection. Our patient population exhibited a significantly unusual immunological pattern: 12% expressed ANAs, 8% anti-heart antibodies, and 10% anti-striated muscle antibodies. These trends became more pronounced in those with complicated myocarditis, with increased rates of 19%, 19%, and 25%, respectively. Anti-muscle and anti-heart antibodies have long been identified using various techniques, but any clinical implications in terms of myocarditis onset remain unclear [15,16]. IFI on the heart section is the classical standard technique for anti-myocardium antibodies detection. However, this technique is not standardised as it can be performed on primate heart and skeletal muscle section, rat heart tissue section using a commercial kit, or in-house, unfixed fresh-frozen cryostat sections of blood group O normal human atrium and skeletal muscle. Apart from the nature of the section, screening serum dilutions are also different, ranging from 1/10 to 1/100 depending on the technique used. Hence, this might account for the huge variation in anti-myocarditis antibodies prevalence, ranging from 12% to 75% compared to 4–34% in control subject, as described in the study conducted by Caforio A.L.P. et al. [15]. In this study, anti-myocardium antibodies were detected in serum samples by IIF at 1/100 dilution on monkey heart and skeletal muscle frozen section, according to manufacturer instructions. Specificity of the assay is high, as 100% of 200 healthy blood donors were negative for anti-myocardium antibodies. Sensitivity was stated as 100% by the manufacturer, but only five myocarditis patients were studied. Hence, we cannot exclude a lack of sensitivity of the technique used for the detection of anti-heart autoantibodies, leading to an underestimation of the prevalence. 

A popular hypothesis suggests that autoantibodies are generated via clonal selection of self-reactive B cell clones triggered by T-cell activation, followed by the release of intracellular or matrix-encrypted proteins when tissue damage develops [17]. However, some such antibodies may play roles in the development of dilated cardiomyopathy [18]. Recent studies have emphasised the adverse prognostic implications of anti-heart antibodies in patients with acute myocarditis [19]. 

In our context, internal medicine consultations were crucial in terms of exploring non-cardiac manifestations and potentially diagnosing AIIDs, although supporting data are limited [20]. Despite such consultations, for most patients, no biological finding afforded a specific diagnosis of an AIID such as lupus, sarcoidosis, or vasculitis; no specific management was initiated. As mentioned above, the only specific diagnosis was one case of Still’s disease. This has prompted us to reconsider the prevalence of AIIDs in patients with myocarditis in an ICCU. Our findings are in agreement with earlier retrospective data: acute myocarditis was the initial manifestation of a later confirmed AIID in only 6.9% of patients [21]. 

However, we observed certain biological phenomena that, to the best of our knowledge, have not been described previously. First, antiphospholipid antibodies were commonly detected on initial assessment of acute myocarditis, with 36% of patients positive for lupus anticoagulant, 7% for anti-B2GP1 antibodies, and 6% for anticardiolipin antibodies. Notably, 25% of patients with controlled lupus anticoagulant levels remained positive after 3 months. No such patient exhibited any other cardiac or non-cardiac manifestation suggestive of antiphospholipid syndrome. As myocarditis does not meet the current diagnostic criteria for that syndrome [22], patient management did not differ from that of the other myocarditis cases.

We explored whether the proportions of CD4+ and CD8+ T-cell subsets might be informative in terms of myocarditis pathogenesis and/or the severity of myocardial damage. This was suggested by the findings of various studies on both murine and human populations [23,24,25]. For example, in a murine model of Coxsackie myocarditis [24], the condition was less severe in CD4+ T-cell-knockout mice. Conversely, in CD8+ T-cell knockout mice with a normal CD4+ T-cell subset, both myocardial infiltration and necrosis were severe, similar to those of the controls. These results suggest that CD4+ T lymphocytes exacerbate myocarditis, and we aimed to provide supportive evidence. In our comprehensive acute myocarditis cohort, i.e., patients along the full disease spectrum, both CD8+ and CD4+ T cell lymphopenia were more common in those with complicated myocarditis. Notably, this T-cell lymphopenia trend seemed to be associated more specifically with CD8+ T lymphocyte status in myocarditis cases who required vasopressors, substantiating the hypothesis that CD8+ T cell lymphopenia serves as a prognostic indicator of an unfavourable clinical course.

Given the absence of any specific diagnosis of myocarditis aetiology in most cases, our treatments were principally cardioprotective drugs and anti-inflammatory medications. The prevailing discharge treatments included beta blockers (100%), ACE inhibitors/angiotensin II receptor blocker/angiotensin receptor, and neprilysin inhibitor (73%), NSAIDs/aspirin (35%), and colchicine (41%). Robust evidence supporting such treatments is lacking for patients who do not exhibit complicated myocarditis. Beta blockers may become recognised as evidence-based therapies; one retrospective study [26] reported poorer outcomes in their absence. It is essential to note that the cited study included primarily myocarditis cases with reduced ejection fractions; thus, caution is essential if extending the findings to other populations. Evidence supporting the prescription of ACE inhibitors/angiotensin II receptor blocker/angiotensin receptor and neprilysin inhibitor for patients with uncomplicated myocarditis is limited or absent. Historically, NSAIDs and aspirin were not prescribed for patients with acute myocarditis because older studies using murine models of myocarditis reported increased mortality [27]. However, more recent studies have alleviated concerns about the use of such drugs. It is now widely accepted that they aid pain management in the absence of any serious safety concern [28]. Colchicine is likely favoured by analogy with pericarditis management and by the findings of the ICAP study [29]. The ongoing ARGO trial assesses the utility of colchicine in patients with complicated myocarditis. Again by analogy with pericarditis, the recent ARAMIS study failed to reveal any benefit of anakinra in an all-comer, acute myocarditis population [30]. 

Our work had several limitations. First, ours was a monocentric cohort, and all patients were enrolled after admission to the ICCU, thereby excluding individuals admitted to wards that potentially treat more patients with AIIDs, such as internal medicine or rheumatology wards. Secondly, the analyses included in the systematic assessment were subjectively chosen, albeit based on the most widely accepted causes of myocarditis, as outlined in a consensus [8]. Third, genetic tests were not performed; the significance of DNA changes became apparent only after patient inclusion commenced. We accept that genetic analyses may well shed valuable light on myocarditis aetiologies [10].

## 5. Conclusions

Data from our thorough systematic etiological assessment of all-comer patients with acute myocarditis admitted to our ICCU were of limited clinical or therapeutic utility. However, the prevalence of antiphospholipid antibodies was high, and such antibodies persisted over time. Anti-heart antibody and T lymphocyte phenotype status may be prognostically useful. Further work is required.

## Figures and Tables

**Table 1:** Patient characteristics.

	All Myocarditis Cases (*N* = 84)	Complicated Myocarditis (*N* = 16)	Uncomplicated Myocarditis (*N* = 68)
Age, mean (years) (Q1–Q3)	34 (22–41)	42 (28–58)	32 (21–38)
Female	18 (21%)	5 (31%)	13 (19%)
Clinical manifestations			
Chest pain	78 (92%)	12 (75%)	66 (97%)
Dyspnoea	14(16%)	6 (38%)	9 (13%)
Flu-like syndrome ^1^	48 (57%)	11 (69%)	37 (54%)
Acute heart failure	6 (7%)	6 (38%)	0 (0%)
Medical history			
Current tobacco use	29 (34%)	5 (31%)	24 (35%)
Previous myocarditis	4 (5%)	1 (6%)	3(4%)
COVID vaccination in the previous 2 weeks	3 (4%)	1 (6%)	2 (4%)
On immune checkpoint inhibitors	4 (5%)	3 (19%)	1 (1%)

^1^ Flu-like syndrome was defined as cough, fever, body aches, and/or headache.

**Table 2:** Initial imaging results.

	All Myocarditis Cases (*N* = 84)	Complicated Myocarditis (*N* = 16)	Uncomplicated Myocarditis (*N* = 68)
Coronary CT	4 (5%)	0 (0%)	4 (6%)
CA	30 (36%)	11 (69%)	19 (28%)
CMRI	83	15 (94%)	68 (100%)
LVEF ^1^, mean (Q1–Q3)	52 (46–60)	42 (30–56)	55 (49–60)
RVEF ^2^, mean (Q1–Q3)	49 (45–53)	45 (40–53)	49 (46–53)
LGE: Number of LV segments, mean (Q1–Q3)	3.8 (2–4)	5.2 (2–9)	3.5 (2–4)
T2 oedema: Number of LV segments, mean (Q1-Q3)	3.6 (2–4)	6.2 (2–10)	3.1 (2–4)
Disease location ^3^			
Anterior	11 (13%)	1 (7%)	10 (15%)
Anterolateral	39 (48%)	4 (27%)	35 (51%)
Inferolateral	52 (63%)	5 (33%)	47 (69%)
Inferior	36 (44%)	6 (40%)	30 (44%)
Inferoseptal	8 (10%)	3 (20%)	5 (7%)
Anteroseptal	10 (12%)	5 (33%)	5 (7%)
Apex	14 (17%)	4 (27%)	10 (15%)
Diffuse disease	5 (6%)	3 (20%)	2 (3%)
Right ventricular involvement	7 (9%)	1 (7%)	6 (9%)

^1^ LVEF: Left ventricle ejection fraction. ^2^ RVEF: Right ventricle ejection fraction. ^3^ Disease location defined by at least one segment with LGE in the respective wall.

**Table 3:** Outcomes and discharge medications.

	All Myocarditis Cases (*N* = 84)	Complicated Myocarditis (*N* = 16)	Uncomplicated Myocarditis (*N* = 68)
Outcomes during the hospital stay			
Ventricular arrythmia ^1^	7 (8%)	3 (19%)	4 (6%)
Supraventricular arrythmia	2 (2%)	1 (6%)	1 (1%)
Use of catecholamines	5 (6%)	5 (31%)	0 (0%)
ECLS ^2^	1 (1%)	1 (6%)	0 (0%)
Death	2 (2%)	2 (13%)	0 (0%)
Discharge medications (*N* = 82)			
Beta blockers	82 (100%)	14 (100%)	68 (100%)
ACE inhibitors/ARB/ARNI ^3^	68 (83%)	14 (100%)	54 (79%)
MRA ^4^	8 (10%)	6 (42%)	2 (3%)
SGLT2i ^5^	8 (10%)	8 (57%)	0 (0%)
NSAIDs ^6^/aspirin	29 (35%)	4 (29%)	25 (37%)
Colchicine	34 (41%)	5 (36%)	29 (43%)
Anakinra	1 (1%)	0 (0%)	1 (1%)
Steroids	3 (4%)	1 (7%)	2 (3%)

^1^ Ventricular arrythmia including non-sustained ventricular tachycardia. ^2^ ECLS: Extracorporeal life support. ^3^ ACE inhibitor: Angiotensin-converting enzyme inhibitors; ARB: Angiotensin II receptor blocker; ARNI: Angiotensin receptor and neprilysin inhibitor. ^4^ MRA: Mineralocorticoid receptor antagonist. ^5^ SGLT2i: Sodium–glucose transport protein 2 inhibitor. ^6^ NSAIDs: Non-steroidal anti-inflammatory drugs.

**Table 4:** Blood panel results.

	All Myocarditis Cases (*N* = 84)	Complicated Myocarditis (*N* = 16)	Uncomplicated Myocarditis (*N* = 68)	*p*-Value
Peak troponin (ng/L)	8337 (1667–19,386)	22,336 (9513–38,598)	6530 (1474–15,526)	0.03
CRP ^1^ mg/L)	31 (11.05–73.6)	65 (17.7–236)	27.8 (10–66.25)	0.03
BNP ^2^ (pg/mL)	50 (28–97)	360 (115–1426)	40 (22–67)	0.008
CK ^3^ (UI/L)	156 (70–459)	466 (152–1387)	116 (68 – 396)	0.04
Haemoglobin (g/dL)	14.1 (13.2–15.0)	13.6 (12.1–14.6)	14.1 (13.2–15.1)	0.11
WBC count ^4^ (G/L)	9.3 (7.3–12.3)	12.3 (8.7–14.7)	9.1 (6.9–11.1)	0.04
Platelet count (G/L)	239 (205–288)	245 (207–280)	236 (205–291)	0.51
Eosinophil count (G/L)	0.06 (0.02–0.19)	0.05 (0.01–0.07)	0.08 (0.03–0.2)	0.75
Ferritin (ng/mL)	220 (135–342)	294 (136–747)	216 (132–322)	0.26

All values except the troponin level were obtained at admission. ^1^ CRP: C-reactive protein. ^2^ BNP: Brain natriuretic peptide. ^3^ CK: Creatinine kinase. ^4^ WBC: White blood cell.

**Table 5:** Immunological systematic panel data.

	All Myocarditis Cases (N = 84)	Complicated Myocarditis (N = 16)	Uncomplicated Myocarditis (N = 68)	*p*-Value
Antinuclear antibodies (*N* = 81)	10 (12%)	3 (19%)	7 (11%)	0.41
Anti-ENA antibodies (*N* = 81)	1 (1%)	0 (0%)	1 (1%)	1
Anti-RNA pol III antibodies (*N* = 81)	1 (1%)	1 (6%)	0 (0%)	0.20
Antiphospholipid antibodies (*N* = 83)				
Lupus anticoagulant				
Positive on admission	30 (36%)	6 (40%)	24 (35%)	0.73
Controlled after 3 months	21	4	17	
Persistently positive but controlled	5 (23%)	0 (0%)	5 (29%)	0.53
Anti-B2GP1 IgM/IgG antibodies				
Positive on admission	1/6 (1%/7%)	0/1 (0%/7%)	1/5 (1%/7%)	1
Controlled after 3 months	3	0	3	
Persistently positive but controlled	0/1 (0%/33%)	NA	0/1 (0%/33%)	
Anticardiolipin IgM/IgG				
Positive on admission	0/5 (0%/6%)	0/0 (0%/0%)	0/5 (0%/7%)	0.58
Controlled after 3 months	4	NA ^1^	4	
Persistently positive but controlled	0/0 (0%/0%)	NA ^1^	0/0 (0%/0%)	
Anti-myocardium (*N* = 83) antibodies	7 (8%)	3 (19%)	4 (6%)	0.11
Anti-striated muscle (*N* = 83) antibodies	10 (12%)	4 (25%)	6 (9%)	0.07
ANCA (*N* = 76)	0 (0%)	0 (0%)	0 (0%)	1

^1^ NA: not applicable.

**Table 6:** T-lymphocyte subset data.

	All Myocarditis (Data Available for 80 Patients)	Complicated Myocarditis (Data Available for 15 Patients)	Non Complicated Myocarditis (Data Available for 65 Patients)	*p*-Value
Total lymphocytes (G/L)	2.08 (1.59–2.85)	1.28 (0.91–2.06)	2.16 (1.7–2.9)	0.001
CD3+ T lymphocytes (G/L)	0.53 (0.38–0.70)	0.45 (0.38–0.53)	0.56 (0.39–0.73)	0.23
T lymphocytes (G/L)	1.59 (1.09–2.183)	1.00 (0.47–1.57)	1.68 (1.23–2.21)	0.001
T lymphocyte proportion (%)	74.6 (68.4–79.4)	72.3 (52.2–78.8)	75.0 (69.9–79.6)	0.09
CD4+ T lymphocytes (G/L)	0.90 (0.67–1.28)	0.56 (0.35–0.82)	1.07 (0.76–1.37)	0.0007
CD4+ T lymphocyte proportion (%)	43.6 (39.1–50.6)	41.1 (33.6–54.0)	44.13 (40.9–50.5)	0.29
CD8+ T lymphocytes (G/L)	0.54 (0.30–0.78)	0.29 (0.11–0.58)	0.57 (0.34–0.79)	0.03
CD8+ T lymphocyte proportion (%)	23.4 (17.3–30.1)	18.0 (10.4–30.7)	24.4 (18.6–29.9)	0.71
CD4+/CD8+ T lymphocyte ratio	2 (1.4–2.7)	2.4 (1.4–2.5)	2 (1.4–2.5)	0.26
Activated T lymphocyte proportion (%)	4.8 (3.4–7.0)	5.0 (3.2–7.4)	4.6 (3.4–6.6)	0.44
Activated CD4+ T lymphocyte proportion (%)	3.7 (2.9–5.5)	3.8 (2.9–5.5)	3.7 (2.9–5.5)	0.29
Activated CD8+ T lymphocyte proportion (%)	5.5 (4.2–9.1)	5.5 (4.1–6.8)	5.3 (4.1–9.1)	0.96

## Data Availability

The datasets that underpin the findings presented in this article can be obtained from the corresponding author upon reasonable request.
